# Multiple Stereoelectroencephalography-Guided Radiofrequency Thermocoagulations for Polymicrogyria With Startle Seizures: A Case Report

**DOI:** 10.3389/fneur.2019.01095

**Published:** 2019-10-18

**Authors:** Yi'Ou Liu, Wenjing Zhou, Bo Hong, Tong Zhao, Chengwei Xu, Jing Ruan, Jianjun Bai, Siyu Wang

**Affiliations:** ^1^Tsinghua University Yuquan Hospital, Beijing, China; ^2^Department of Biomedical Engineering, School of Medicine, Tsinghua University, Beijing, China; ^3^Inner Mongolia People's Hospital, Inner Mongolia Autonomous Region, Hohhot, China

**Keywords:** multiple SEEG-guided RF-TCs, polymicrogyria, startle seizure, stereoelectroencephalography (SEEG), surgical outcome

## Abstract

The best results of stereoelectroencephalography (SEEG)-guided radiofrequency thermocoagulation (RF-TC) were observed in epilepsies with more limited lesions, but this procedure is rarely used in a wide range of brain malformation. We report a rare case of polymicrogyria (PMG) combined with drug-resistant startle seizures. Presurgical monitoring was performed using SEEG owing to the large lesion and complexity of PMG. According to the intracranial electrode results, the seizure onset was extensive, with the onset starting earlier in the cingulate sulcus and insular pole than in other sites of the other electrodes. Multi-point and multi-step SEEG-guided RF-TC was used for diffuse lesion and functional protection. RF-TC was first applied to the cingulate sulcus and insular pole, and our patient was rendered free from startle seizures after 2 weeks. Two weeks of observation helped us to observe the efficacy of RF-TC and the changes of SEEG, so as to make the next TC scheme. The patient still had spontaneous seizures after the first treatment. RF-TC was then applied to other sites involved earlier. Finally, the patient reached Engel class IIa for a follow-up period of 1 year. There were no additional startle seizures, and important functional areas were protected.

## Introduction

Clinically, it is rare for polymicrogyria (PMG) to combine with startle seizures, and PMG itself can induce epilepsy (1). Previously reported cases of startle seizures were mostly located in the inner side of the frontal lobe, and they may overlap with cortical malformation. In one reported case of PMG with startle seizures, it was found that the region involved in startle seizures was the lateral part of the perirolandic region rather than the supplementary motor area (SMA), although SMA regions are widely accepted to be associated with startle seizures (2). This is partly because of different organizations of the network caused by cortical malformation (1). Stereoelectroencephalography (SEEG)-guided radiofrequency thermocoagulation (RF-TC) is a safe stereotactic invasive treatment of drug-resistant epilepsy. A recent study found that RF-TC had higher seizure control rate related to three well-limited malformations: hypothalamic hamartomas, periventricular nodular heterotopias, and focal cortical dysplasias (3). In a meta-analysis of 296 patients, four etiologies were assessed for efficacy, and the greatest efficacy was observed in patients with periventricular nodular heterotopia (4). French guidelines on SEEG also suggested that SEEG-guided RF-TC may be an alternative to conventional surgery when the epileptogenic zone (EZ) has been proved to be very focal (5). We present here a rare case of PMG with startle seizures. Lesions on both the image and seizure onset were diffuse, which seemed difficult to be treated with RF-TC. A multicentric study analyzed 58 cases with PMG-related epilepsy. Six patients underwent RF-TC. Only two cases with relatively limited SEEG-delineated EZ improved (6). A recent review showed that none of the patients with PMG with diffuse onset achieved seizure freedom after surgery (7). In addition, PMG may retain some functions; a large resection would probably result in functional deterioration (6–8); thus, a multi-point and multi-step TC procedure was applied to maximize the damage to the epileptic focus while protecting the patient's functions. It has been reported that number of intralesional RF-TC sites was the main predictor of good outcome (9). And multiple RF-TCs can be used as a palliative option for discrete lesion within the epileptic network (10). We continued SEEG recordings for 2 weeks after first RF-TC. This procedure could help us to verify different hypotheses according to efficacy, and a decision on the next TC scheme could be made. The patient's startle seizures were stopped, and the postsurgical patient reached Engel IIa. The patient's quality of life was improved, and her functions were effectively protected.

## Case Study

A 27-year-old left-handed female with an 8-year history of drug-resistant epilepsy was referred to our center for presurgical evaluation. Eight years ago, the patient suddenly fell while going upstairs, and it was suspected that this might have been caused by “congenital heart disease.” At that time, atrial septal defect repair was performed in a local hospital. After surgery, there was a good recovery of heart function. However, the seizures were not alleviated. The patient presented focal seizures characterized by a sudden sensation of stiffness and rigidity of the right limb (primarily the right lower limb) with confusion lasting for more than 10 s; she often falls to ground and is frequently injured. The seizures were mostly provoked by sudden unexpected events, primarily loud sounds, with a frequency of three to five times per week. Her seizures were resistant to multiple medications, including sodium valproate, levetiracetam, lamotrigine, and clonazepam. She was taking lamotrigine, 300 mg/day, and levetiracetam, 1,500 mg/day. The clinical examination revealed mild hemiplegia of the right side, although she could walk and run. She could elevate her right upper limb to shoulder height but without any useful finger function. Her speech function was unaffected.

## Non-Invasive Workup

**Figure 1:**
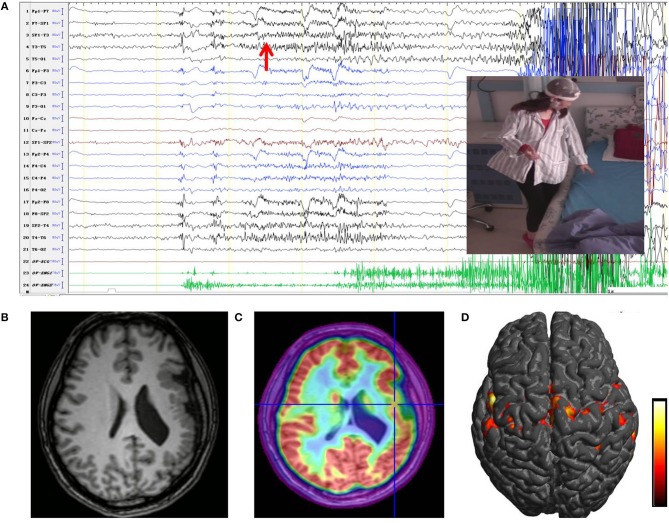
**(A)** Ictal EEG originated in the left unilateral PMG. The first ictal modifications were characterized by left frontotemporal rhythmic fast activity (red arrow), followed by a focal left temporal alpha rhythm. Ictal clinical signs shown in the image corresponded to sudden sensation of stiffness and rigidity of the right limb; the patient was unable to stand stably. **(B)** An MRI scan showed a multi-lobar PMG involving the left frontal lobe, temporal lobe, insula, and temporo-parieto-occipital junctions. **(C)** PET/MRI imaging co-register of the patient showing significant hypometabolism in the middle part of the PMG. **(D)** Results of fMRI showed a significant increase in BOLD signal over the left PMG when moving the right leg, especially the perisylvian area (from red to yellow, the BOLD signal intensity increased). PMG, polymicrogyria; BOLD, blood oxygen level dependent.

Video electroencephalography (VEEG) monitoring showed normal background with interictal activities in the left anterior part of head. Seven clinical seizures were recorded during VEEG sessions with the above-described semiology, with and without a startle component. Seizure onset was characterized by rapid rhythmic spikes on the left anterior and medial temporal lobes, with maximum in T3 (Figure 1). An MRI scan demonstrated the left PMG (Figure 1). Hypometabolism on FDG-PET pointed to the middle part of the PMG.

MRI protocol included three-dimensional (3D) T1-weighted 1.0 mm thick contiguous slices. Fluid-attenuated inversion recovery (FLAIR) sequences, double inversion recovery (DIR) sequences, and T2-weighted (2D, axial and coronal) and FLAIR sequences (2D, axial and coronal) were also obtained. FDG-PET was superimposed on the 3D brain MRI scans to determine the potential alterations of metabolism (Figure 1). Functional MRI (fMRI) was performed on the patient. Blood oxygen level-dependent (BOLD) signal changes were sequentially analyzed using SPM12 in the program MATLAB (11). The fMRI pattern revealed bilateral motor and language representation (Figure 1).

## Invasive Procedures

**Figure 2:**
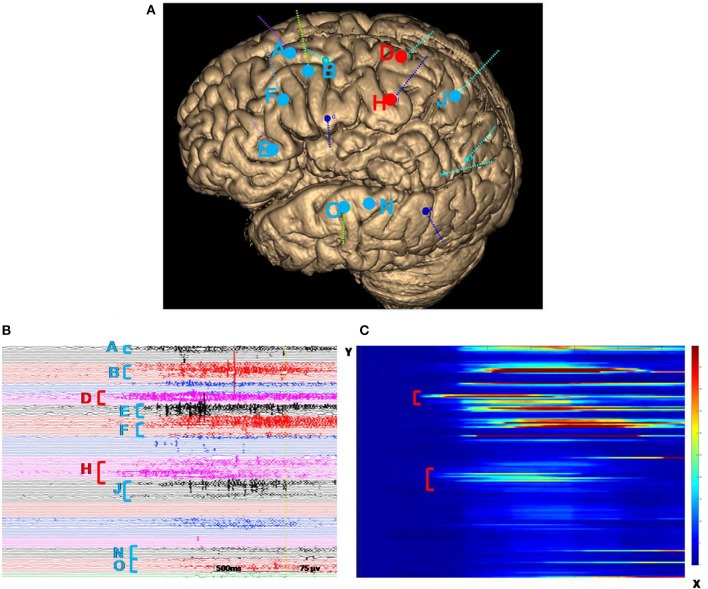
**(A)** A three-dimensional MRI reconstruction of the depth electrodes. **(B)** Ictal SEEG recording revealed that the first electrodes involved in the seizure were from the cingulate sulcus and insular pole (electrodes D and H, marked by red vertical bars). Then the midcingulate cortex, inferior part of the insula, anterior short gyrus, anterior part of the insula, precuneus, superior temporal gyrus, and operculum quickly became involved (electrodes A, B, E, F, J, N, and O, marked by blue vertical bars). The color of the letters is consistent with the dots in illustration A. **(C)** The *x*-axis represents the time of the selected seizure; the *y*-axis indicates electrodes. The energy ratio (ER) of high-frequency changes was depicted in a color scale (from red to blue, the ER intensity decreased). Quantification of the seizure was consistent with visual analysis, indicating that the cingulate sulcus and insular pole (marked in red) were involved first. SEEG, stereoelectroencephalography.

We decided to proceed with intracranial electrode implantation in this patient because the precise position involved first during the seizures was not indicated, although imaging, symptomatology, and VEEG were all consistent regarding the lesion of the left PMG. Additionally, the PMG involved multiple lobes and functional areas. SEEG was proposed to identify the EZ and better define a possible surgical resection limit. A total of 14 electrodes (8–16 contacts; length: 2 mm; diameter: 0.8 mm; 1.5 mm apart) were implanted in stereotactic conditions covering almost all areas of the PMG and other suspected regions beyond lesions to explore the epileptogenicity of brain structures (12, 13). SEEG was performed over 10 days in the Epilepsy Center at Tsinghua University Yuquan Hospital in Beijing, China, using a 256-channel Neurofax software (MEE-1000; Nihon Kohden, Tokyo, Japan) system. Once the monitoring was completed, SEEG data were reviewed by the epileptologist to identify the EZ and early propagation. The interictal recordings showed a wide range of irritative areas involving the majority of the left PMG especially the anterior part, consisting of slow waves and spikes. Thirteen clinical seizures were recorded, among which eight seizures were provoked by sudden unexpected events, primarily loud sounds. We defined the EZ by visual analysis assisted by a quantitative method (epileptogenicity index) (14). The initial involvement of the seizures was extensive and characterized by low-voltage fast activity originating from regions both inside and outside the PMG, with the onset starting earlier in the cingulate sulcus and insular pole than in the locations of the other electrodes (Figure 2). At the onset of the seizure, 43 contacts were involved in the region of the PMG, and nine contacts were involved outside the region of the PMG. EZ involved 82.7% of total PMG. The signal analysis of fast activities (defined by a high epileptogenicity index [≥0.4]) showed prominent early activation involving the cingulate sulcus and insular pole (Figure 2). Functional stimulations were performed with a clinical stimulator (MEE-1000; Nihon Kohden, Tokyo, Japan) to reproduce the clinical symptomatology and to localize functional areas that must be retained during surgery (15, 16). Bipolar Electrical Stimulation (ES) was carried out at 50 Hz (pulse width 1 ms, delivered for 5 s). The stimulus intensity started at 3 mA and increased in 1- to 2-mA steps up to 15 mA (17). When ES with an electrode reproducibly induced language production disturbances—that is, speech arrest, significant slowing of speech, paranomia, or perseveration or comprehension impairments—in at least two independent trials, the site of this electrode was indicated to be positive. Cortical mapping was performed by recording the clinical symptoms induced by stimulation (18). When stimulating electrodes D, E, H, J, K, and L (electrodes shown in Figure 2), the patient showed signs of right limb sensation and movement.

## Radiofrequency Thermocoagulation

**Figure 3:**
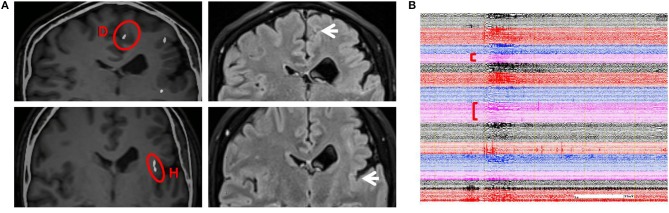
**(A)** Illustration on the left shows early-onset electrode D (contacts 1–5) and H (contacts 9–15) in which RF-TC was performed first (highlighted with red circles). Illustration on the right shows sites of coagulation in the same region on an MRI image acquired 6 months after the first RF-TC (arrows on the right). The patient was free from startle seizures even when provoked by sudden unexpected sounds or other events during the following 2 weeks of monitoring. **(B)** The patient did not have a seizure when stimulated by a sudden unexpected sound; SEEG showed a similar pattern of a startle-induced seizure, yet there was no trace of discharges in electrodes D and H after RF-TC. RF-TC, radiofrequency thermocoagulation.

**Figure 4:**
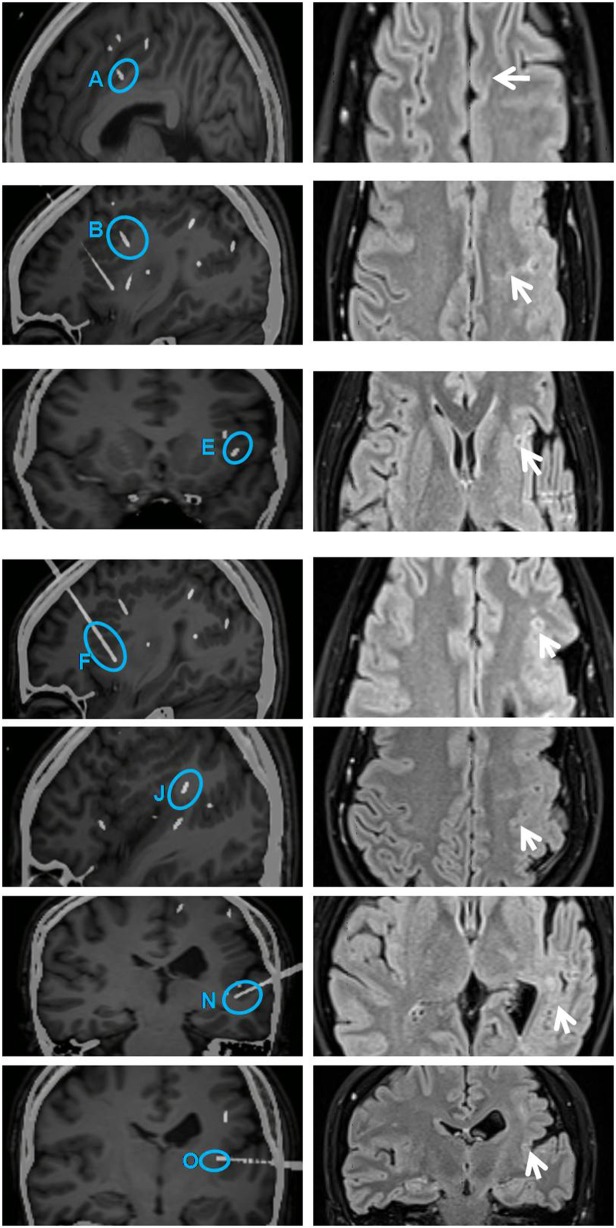
Illustration on the left shows secondarily involved electrodes A (contacts 1 and 2), B (contacts 1–10), E (contacts 1–4), F (contacts 1–9), J (contacts 3–10), N (contacts 1–5), and O (contacts 1 and 2) (highlighted with blue circles). All these contacts were subjected to RF-TC 2 weeks after the first thermocoagulation. Illustration on the right shows sites of coagulation in the same region on an MRI image acquired 6 months later (arrows on the right). The diameter of lesions ranged from 4 to 5 mm. RF-TC, radiofrequency thermocoagulation.

From SEEG data, fMRI, and intracranial ES results, it could be seen that EZ originated from multiple lobes, and the PMG region still retained its function. It means EZ overlaps with functional areas, and removing this part would result in new neurological deficits. Therefore, multiple RF-TCs were the reasonable treatment to avoid functional damage. The technical details of RF-TC have been provided previously (10, 19–21). Since 2014, RF-TC has been provided to every patient receiving SEEG implantation at our epilepsy center. Electrodes should cover as much of the PMG as possible, not only to assess epileptogenicity in different areas but also to destroy as many lesions as possible (22). Pertinent contacts were connected to generator equipment (GN300, Aesculap AG, Germany), which was modified for SEEG electrodes. Thermolesions were performed without anesthesia. We have adopted the following parameters: the current power from 1.5 W gradually increased until the power delivered by the generator collapsed (usually within a few seconds); the maximal lesion volume may be reached by using parameters adjusted according to the impedance. Then we recorded the discharge of the relevant electrode contacts, and the epileptiform activity disappeared after TC (23). Areas within 2 mm of the vascular structure and areas where clinical symptoms are induced by ES should be kept away from TC (24). A total of 52 contacts were performed. The location of RF-TCs is usually determined by the electrical change at the early stage of the seizure, usually manifested as low-voltage fast activity. From SEEG data, although there was a wide range of EZs, the onset starting earlier in the cingulate sulcus and insular pole than in the locations of the other electrodes; therefore, RF-TC was first applied in the cingulate sulcus (D, contact 1–5) and insular pole (H, contact 9–15) (Figure 3), which suppressed the fast activities within electrodes D and H (Figure 3) and rendered the patient free from startle seizures after 2 weeks of observation. Because the patient still had frequent unprovoked seizures represented by low-voltage fast activities in multiple regions as seen from the seizure data of SEEG, RF-TC was then applied in the midcingulate cortex, inferior part of the insula, anterior short gyrus, anterior part of the insula, precuneus, superior temporal gyrus, and operculum (Figure 4).

## Seizure Outcome

Post-RF-TC outcome was evaluated 3 months, 6 months, and 1 year after the procedure. The patient's startle seizures completely disappeared, and she reached Engel class IIa in the postsurgical process; she had a total of five seizures within 1 year after RF-TC (a reduction of 95% in seizure frequency). No unexpected, severe permanent neurological morbidity or cognitive impairment occurred after RF-TC. No local pain or brain swelling around the coagulated region developed during coagulation of regions. No complication related to RF-TC procedures has been found. Postoperative MRI scans were performed 6 months after RF-TC in order to evaluate the anatomic extent of the lesion. Multiple coagulative necrosis was shown along the electrode trajectories. The diameter of lesions ranged from 4 to 5 mm.

## Discussion

PMG is one of the most common and refractory cortical developments (MCDs) (25), making up nearly 20% of all MCDs. PMG is highly epileptogenic (26), and about 80% of patients develop medically refractory epilepsy eventually. In a recent multicenter study, in the 143 surgical cases of epilepsy, only 5% were MCD-related epilepsy (27), and in 9,523 patients with epilepsy, only 0.8% of patients had histopathological diagnosis of PMG and received surgical treatment (28, 29). Some difficulties exist in the surgical treatment of PMG. (1) The localization of EZ: the lesion of PMG is usually bilateral or multi-lobar, and the EZ may be within or outside the abnormality (6). (2) Functional protection: PMG can be both epileptogenic and functional, and removal of malformations can result in functional impairment (30). In previous cases, invasive intracranial electrodes were used to search for the EZ in PMG, and good surgical outcome was achieved (31). Nevertheless, some patients were excluded from surgery owing to functional reasons. Patients who underwent hemispherectomy or perirolandic resections developed new postoperative deficits. Even after SEEG implantation, an extensive resection of the lesion may result in severe functional impairment. Seizure semiology of PMG often reflected PMG location; coughing and vomiting are common manifestations with involvement of the perisylvian region (8).

Startle epilepsy is a clinically rare form of epilepsy. A characteristic of startle epilepsy is a sudden, unanticipated seizure, usually triggered by unexpected stimuli. Patients often have large lesions, such as perinatal hypoxia injury involving the hemisphere (2). Accordingly, hemispherectomy and hemisphere dissection are commonly used to treat startle seizures. A number of early studies and case reports have suggested that startle epilepsy is associated with SMA involvement (31, 32).

For our patient, PMG involved multiple lobes, including the SMA, which may be highly associated with startle seizures. SEEG evaluation was judged to be necessary to find EZs in PMG and better define a possible surgical resection limit. After SEEG implantation, this patient had a wide range of seizure onsets originating from regions both inside and outside the PMG, suggesting that there may be epileptogenic areas or functional impairment beyond the structural abnormality. In particular, the first part of the brain network to be involved after the startle seizures was located in the cingulate sulcus outside the PMG, which is not in the region of the SMA that we have traditionally thought of in relation to startle seizures, proving the heterogeneity of PMG-induced epilepsy and the complexity of the epileptic network. Thus, for PMG, we cannot ignore the epilepsy-inducing area outside the lesion. Furthermore, we should protect function to the largest extent, as there is evidence that the polymicrogyric cortex usually retains functions (31).

The best results of RF-TC were observed in epilepsies with more strictly limited lesions. A significant seizure frequency reduction was observed in patients with periventricular nodular heterotopias, hypothalamic hamartomas, and focal cortical dysplasias (4). TC is rarely used in a wide range of brain malformations. Some of these patients are not suitable for surgery after SEEG because EZs were widespread or located close to functional areas. However, some authors suggested that enhancing the number of RF-TC sites for delineating the epileptogenic area resulted in long-lasting seizure freedom (33), the number of intralesional RF-TC sites was the main predictor of good outcome (31), and multiple RF-TCs can be used as a palliative option targeting crucial nodes within the epileptic network, in order to interrupt the seizure propagation (34). It would be a surgical technique for discrete or diffuse epileptogenic lesions, and the number of thermocoagulations is determined by the size of the targeted zone and the network targeted (10). We applied this multi-point TC procedure to maximize the damage to the epileptic focus while protecting the patient's motor and language functions. The use of the same electrode for RF-TC has the additional advantage of eliminating the risk of further implantation of the electrodes (19, 35). PMG changes the normal brain network, the symptoms are diverse, and the EZs are difficult to locate. Meanwhile, the function of the affected region is retained. Accordingly, electrode implantation was designed for extensive coverage of the PMG, not only to assess epileptogenicity in different areas but also to destroy as many lesions as possible, which enabled us to have good surgical effect. In a larger case series of epilepsy surgery related to PMG, none of the patients with diffuse seizure onset achieved seizure freedom after surgery (9, 36). Our results indicated that patients who had a wide range of brain malformations could be considered for this treatment. Such a strategy could be an option in large-sized lesions for which resective surgery seems risky. From the results of the surgery, it may be hypothesized that seizures of PMG may be caused by part of the malformation, whose destruction may disconnect the epileptic network. Multiple RF-TCs within such a network could lead to disruption of epileptogenic network, thereby causing a significant seizure reduction (10). Because this patient had diffuse lesion, parameters of radiofrequency current can be adapted to optimize the size of the lesion. Recent work showed that the largest possible lesions produced when radiofrequency power was applied for long duration at <3 W (20). We adopted the current power from 1.5 W and gradually increased it until the impedance suddenly increases; this is related to the coagulation of proteins producing a sudden modification of the resistance. This approach has been compared with the parameters used in the empiric procedure and is superior in size optimization (20). No permanent neurological or cognitive impairment has occurred in most cases, with some cases showing transient adverse effects such as mouth dysaesthesia, motor apraxia, or a transient local pain during procedure, which disappeared several days or months later (23). Rare cases have shown unexpected major permanent neurologic deficits (19).

In addition to multi-point TC, a time window was also added for observation. Thus, the efficacy could be observed, and a decision on the next TC scheme could be made. In our previous report, the SEEG electrodes were often removed at the end of the RF-TC, and the patient was discharged 24 h after TC (9, 23), Some authors suggested to leave the electrodes in place after the RF-TC and analysis of the SEEG data are continued, which can help us to verify different hypotheses and make plans for the next treatment (37). French guidelines on SEEG pointed out that continued SEEG recording can be useful after SEEG-guided RF-TC. More and more attention has been paid to RF-TC as a diagnostic tool when it is difficult to differentiate multiple hypotheses in complex situations; the effect of the RF-TC could be very informative and could lead to a better understanding of the epileptic network (10). The latest opinion also showed that it is reasonable to continue the SEEG for a few more days after RF-TC, and no hemorrhagic complications have been reported. But no exact time window has been mentioned. In this patient, we adopted a 2-week efficacy observation on the basis of her seizure frequency, which could help us to observe the efficacy of first RF-TC and the changes of SEEG. Thus, a decision on the next TC scheme could be made. The onset of the seizures was in the cingulate sulcus and insular pole; these sites underwent TC first. In the subsequent 2 weeks of monitoring, the patient did not have a startle seizure. Earlier studies reported that startle seizures are associated with SMA. However, we found no early electrical activities in the SMA region; the SMA was only found to be involved in the early propagation region. Recent studies have shown that EZ involved in startle seizures extends further than expected, and networks are more widespread than we thought, especially in the presence of developmental abnormalities, which include the frontal motor/premotor cortex, insula, precuneus, and primary sensory cortex (2). This is consistent with our results, yet early propagation of SMA suggests that it is also involved in brain networks associated with startle seizures (23). Whether the cingulate sulcus activated the insular pole or vice versa cannot be verified. The initiation of the insular pole has not been seen in studies. After 2 weeks of observation, the patient had non-startle-induced seizures. The onset pattern was similar to the previous startle seizure pattern, yet the discharges of electrodes D and H disappeared (Figure 3). This patient's remaining spontaneous seizures told us that we may not completely interrupt the epilepsy network after first treatment, or we just blocked the epileptic triggers associated with startle seizure. Therefore, we carried out a secondary multi-point TC for the early propagation regions (Figure 4). The patient finally achieved a good curative effect. We would like to emphasize that the proposed 2-week observation period is relatively subjective, as factors such as seizure frequency and tolerance of different patients should also be considered.

## Conclusion

We applied a surgical strategy to treat a rare case of combined PMG and startle seizures, with EZs located close to the cortex with high functional value and poorly accessible to conventional surgical procedures (33). After the surgery, the patient reached Engel class IIa, the startle seizures completely disappeared, and the functions were protected. Accordingly, multiple SEEG-guided RF-TC should be applied for drug-resistant epilepsy with extensive malformation of cortical development (1). This procedure has been accepted as a first therapeutic step before a resection surgery, it is relatively safe, and it can achieve palliative results when surgery is not possible.

## Ethics Statement

This study was approved by the ethics committee of Tsinghua University Yuquan Hospital. Written informed consent was obtained from the patient for the publication of this case report and the accompanying image. This is separate from the patient's consent to participate in the treatment.

## Author Contributions

Y'OL wrote the main manuscript text and analyzed the data, while WZ participated in the design and coordination of the study and has been involved in revising the manuscript for important intellectual content. BH and TZ performed EI data analysis and interpretation. SW was responsible for psychological report. Other members are responsible for data collection. All authors have read and approved the content, and agree to submit for consideration for publication in the journal.

### Conflict of Interest

The authors declare that the research was conducted in the absence of any commercial or financial relationships that could be construed as a potential conflict of interest.
